# Noncanonical Role for the Host Vps4 AAA+ ATPase ESCRT Protein in the Formation of *Tomato Bushy Stunt Virus* Replicase

**DOI:** 10.1371/journal.ppat.1004087

**Published:** 2014-04-24

**Authors:** Daniel Barajas, Isabel Fernández de Castro Martín, Judit Pogany, Cristina Risco, Peter D. Nagy

**Affiliations:** 1 Department of Plant Pathology, University of Kentucky, Lexington, Kentucky, United States of America; 2 Cell Structure Laboratory, Centro Nacional de Biotecnología (CNB-CSIC), Campus de Cantoblanco, Madrid, Spain; University of California Riverside, United States of America

## Abstract

Assembling of the membrane-bound viral replicase complexes (VRCs) consisting of viral- and host-encoded proteins is a key step during the replication of positive-stranded RNA viruses in the infected cells. Previous genome-wide screens with *Tomato bushy stunt* tombusvirus (TBSV) in a yeast model host have revealed the involvement of eleven cellular ESCRT (endosomal sorting complexes required for transport) proteins in viral replication. The ESCRT proteins are involved in endosomal sorting of cellular membrane proteins by forming multiprotein complexes, deforming membranes away from the cytosol and, ultimately, pinching off vesicles into the lumen of the endosomes. In this paper, we show an unexpected key role for the conserved Vps4p AAA+ ATPase, whose canonical function is to disassemble the ESCRT complexes and recycle them from the membranes back to the cytosol. We find that the tombusvirus p33 replication protein interacts with Vps4p and three ESCRT-III proteins. Interestingly, Vps4p is recruited to become a permanent component of the VRCs as shown by co-purification assays and immuno-EM. Vps4p is co-localized with the viral dsRNA and contacts the viral (+)RNA in the intracellular membrane. Deletion of Vps4p in yeast leads to the formation of crescent-like membrane structures instead of the characteristic spherule and vesicle-like structures. The *in vitro* assembled tombusvirus replicase based on cell-free extracts (CFE) from *vps4Δ* yeast is highly nuclease sensitive, in contrast with the nuclease insensitive replicase in wt CFE. These data suggest that the role of Vps4p and the ESCRT machinery is to aid building the membrane-bound VRCs, which become nuclease-insensitive to avoid the recognition by the host antiviral surveillance system and the destruction of the viral RNA. Other (+)RNA viruses of plants and animals might also subvert Vps4p and the ESCRT machinery for formation of VRCs, which require membrane deformation and spherule formation.

## Author Summary

Replication of positive-stranded RNA viruses depends on recruitment of host proteins and cellular membranes to assemble the viral replicase complexes. Tombusviruses, small RNA viruses of plants, co-opt the cellular ESCRT (endosomal sorting complexes required for transport) proteins to facilitate replicase assembly on the peroxisomal membranes. The authors show a surprising role for the ESCRT-associated Vps4p AAA+ ATPase during tombusvirus replication. They show that Vps4p is recruited to and becomes a permanent member of the replicase complex through its interaction with the viral replication proteins. Also, EM and immuno-EM studies reveal that Vps4p is required for the formation of single-membrane vesicle-like structures, called spherules, which represent the sites of tombusvirus replication. The authors propose that Vps4p and other ESCRT proteins are required for membrane deformation and replicase assembly.

## Introduction

Plus-stranded (+)RNA viruses replicate by assembling membrane-bound viral replicase complexes (VRCs) consisting of viral- and host-coded proteins in combination with the viral RNA template in the infected cells. Although major progress has recently been made in understanding the functions of the viral replication proteins, including the viral RNA-dependent RNA polymerase (RdRp) and auxiliary replication proteins, the contribution of many host proteins to VRC assembly is far from complete [1]–[7]. The host proteins contributing to VRC assembly likely include translation factors, protein chaperones, RNA-modifying enzymes, and cellular proteins involved in lipid biosynthesis [8]–[14]. Other host proteins, such as the ESCRT proteins, reticulons and amphiphysins could be involved in membrane deformation occurring during VRC assembly [15]–[17]. However, the actual functions of the majority of the identified host proteins involved in VRC assembly have not been fully revealed.

To assemble their VRCs, RNA viruses take control of cell membranes by interfering with intracellular lipid metabolism, protein regulation, targeting and transport [7], [18]. Viral polymerases of many (+)RNA viruses interact with membranes and build functional VRCs in spherules that are single-membrane vesicles with a narrow opening to the cytosol. Spherules form as invaginations in a variety of cell organelles [7], [18], [19]. Tubulovesicular cubic membranes, double membrane vesicles (DMV) and planar oligomeric arrays are some other classes of membranous structures that can harbor VRCs as documented in the literature [18].

TBSV is a small (+)RNA virus that has recently emerged as a model virus to study virus replication, recombination, and virus - host interactions using yeast (*Saccharomyces cerevisiae*) as a model host [7], [20]–[23]. Several systematic genome-wide screens and global proteomics approaches have led to the identification of ∼500 host proteins/genes that interacted with the viral replication proteins or affected TBSV replication and recombination [9], [11], [24]–[32]. Subsequent detailed analysis revealed the functions of the two viral replication proteins (i.e., p33 and p92^pol^), the viral RNA, the host heat shock protein 70 (Hsp70) and the eukaryotic elongation factor 1A (eEF1A), sterols and phospholipids in the assembly of the tombusvirus VRCs [30], [31], [33]–[41]. Hsp70 and eEF1A proteins have been shown to bind to the viral replication proteins [1], [33], [42]. The auxiliary p33 replication protein, which is an RNA chaperone, recruits the TBSV (+)RNA to the site of replication, which is the cytosolic surface of peroxisomal membranes [43]–[48]. The RdRp protein p92^pol^ binds to the essential p33 replication protein that is required for assembling the functional VRC [22], [34], [45], [49], [50].

Interestingly, the genome-wide screens and a proteome-wide over-expression approach for host factors affecting TBSV replication in yeast [9], [11], [26] have led to the identification of 11 ESCRT (endosomal sorting complexes required for transport) proteins involved in multivesicular body (MVB)/endosome pathway [51], [52]. The identified host proteins included Vps27p (ESCRT-0 complex); Vps23p and Vps28p (ESCRT-I complex), Vps25p and Vps36p (ESCRT-II complex); Snf7p and Vps24p (ESCRT-III complex); Doa4p ubiquitin isopeptidase, Did2p having Doa4p-related function; Bro1p ESCRT-associated protein and Vps4p AAA+ ATPase [9]. The identification of ESCRT proteins led to a model that tombusvirus replication depends on hijacking of ESCRT proteins to the peroxisomal membrane. It has been suggested that the protection of the viral RNA is compromised within the VRC assembled in the absence of cellular ESCRT proteins. Altogether, the formation of membranous spherule-like replication structures in infected cells might require co-opted ESCRT proteins. However, the actual functions of the subverted ESCRT proteins in TBSV replication are currently unknown.

A set of 20–30 ESCRT proteins is important for the endosomal/multivesicular body (MVB) protein-sorting pathway in eukaryotic cells, which down-regulates plasma membrane proteins via endocytosis; and sorts newly synthesized membrane proteins from trans-Golgi vesicles to the endosome, lysosome or the plasma membrane [53]–[55]. The ESCRT proteins are involved in membrane invagination and vesicle formation during the MVB pathway. Defects in the MVB pathway can cause serious diseases, including cancer, early embryonic lethality and defect in growth control [53]–[56]. Also, enveloped retroviruses (such as HIV), (+) and (−)RNA viruses (such as filo-, arena-, rhabdo- and paramyxoviruses) redirect cellular ESCRT proteins to the plasma membrane, leading to budding and fission of the viral particles from infected cells [51], [52].

The MVB pathway starts with the recognition of monoubiquitinated cargo proteins in the endosome by Vps27p (ESCRT-0 complex), which serves as a signal for proteins to be sorted into membrane microdomains of late endosomes [54]–[57]. Vps27p in turn recruits Vps23-containing ESCRT-I complex and then the ESCRT-II complex, resulting in grouping the cargo proteins together in the limiting membranes of late endosomes and deforming the membranes that leads to membrane invagination into the lumen [58], [59]. Then, components of the large ESCRT-III complex are recruited from the cytosol, followed by sequential assembly of ESCRT-III monomers into helical lattice on the membrane that leads to the scission of the neck of the invaginated membrane, giving raise to vesicle budding into the lumen of endosome and to MVB formation [60], [61]. Then, Vps4p recycles the ESCRT proteins, whereas Doa4p recycles the ubiquitin, leading to budding of multiple small vesicles into the lumen [57].

The ESCRT-III and the Vps4p AAA+ ATPase together comprise a conserved membrane scission machinery. The ATP-dependent function of Vps4p is to disassemble and remove the ESCRT-III components from the membranes (i.e., recycling them back to the cytosol) [59], [62]. Vps4p is a member of the AAA+ ATPase family, which uses ATP to remodel macromolecular structures in various biological processes, such as protein disaggregation, microtubule severing and membrane fusion. The N-terminal part of Vps4p, termed MIT domain, binds to the ESCRT-III components, while the ATPase domain is involved in ATP hydrolysis. Vps4p is present as an inactive dimer in the cytosol and during activation, Vps4p likely forms a dodecamer with two parallel rings and the ESCRT-III components are likely pulled across the central hole of these rings during the Vps4p-driven recycling event [62].

Our previous works have demonstrated that tombusviruses co-opt selected components of the cellular ESCRT machinery for replication via interaction of the p33 replication protein with Vps23p (similar to Tsg101 in mammals) and Bro1p (ALIX) [15], [63]. The recruitment of these cellular ESCRT proteins to the sites of tombusvirus replication is assumed to lead to the sequential recruitment of additional selected ESCRT factors, such as ESCRT-III and Vps4p AAA+ ATPase. Indeed, in the absence of these cellular factors in yeast or the expression of dominant negative versions of these proteins in *N. benthamiana* host plant, tombusvirus replication is decreased by 10-to-20-fold [15], [63]. Based on the known functions of the ESCRT proteins, it was suggested that the ESCRT proteins are recruited by TBSV to aid the formation of VRCs, which require membrane deformation to induce spherule-like structures. However, the presented data do not explain how the “neck” of the spherule-like replication structure is stabilized (to maintain an opening towards the cytosol) and why the recruitment of ESCRT factors does not lead to enclosure of the replicase complex (i.e., the VRCs are not converted rapidly into vesicles via scission of the necks of the spherules that bud inside the peroxisomes). The latter event is predicted if TBSV would take advantage of the canonical functions of the ESCRT proteins, which always result in budding of the newly formed vesicles away from the cytosol [57], [59].

In this paper, we identify Vps4p AAA+ ATPase as a major host factor in TBSV replication. We show that the p33 replication protein interacts with Vps4p and three other ESCRT-III proteins. Surprisingly, we find that Vps4p is a permanent member of the tombusvirus VRCs and it also interacts with the viral RNA. EM images revealed that Vps4p is localized in a compartment also containing the viral double-stranded (ds)RNA. Altogether, we propose that the interaction of p33 and Vps4p is critical for spherule formation and efficient tombusvirus replication. These data are consistent with the model that TBSV co-opts ESCRT proteins for its replication and these ESCRT proteins play noncanonical functions in aiding VRC formation and TBSV replication.

## Results

### Vps4p AAA+ ATPase is a component of the tombusvirus replicase

**Figure 1:**
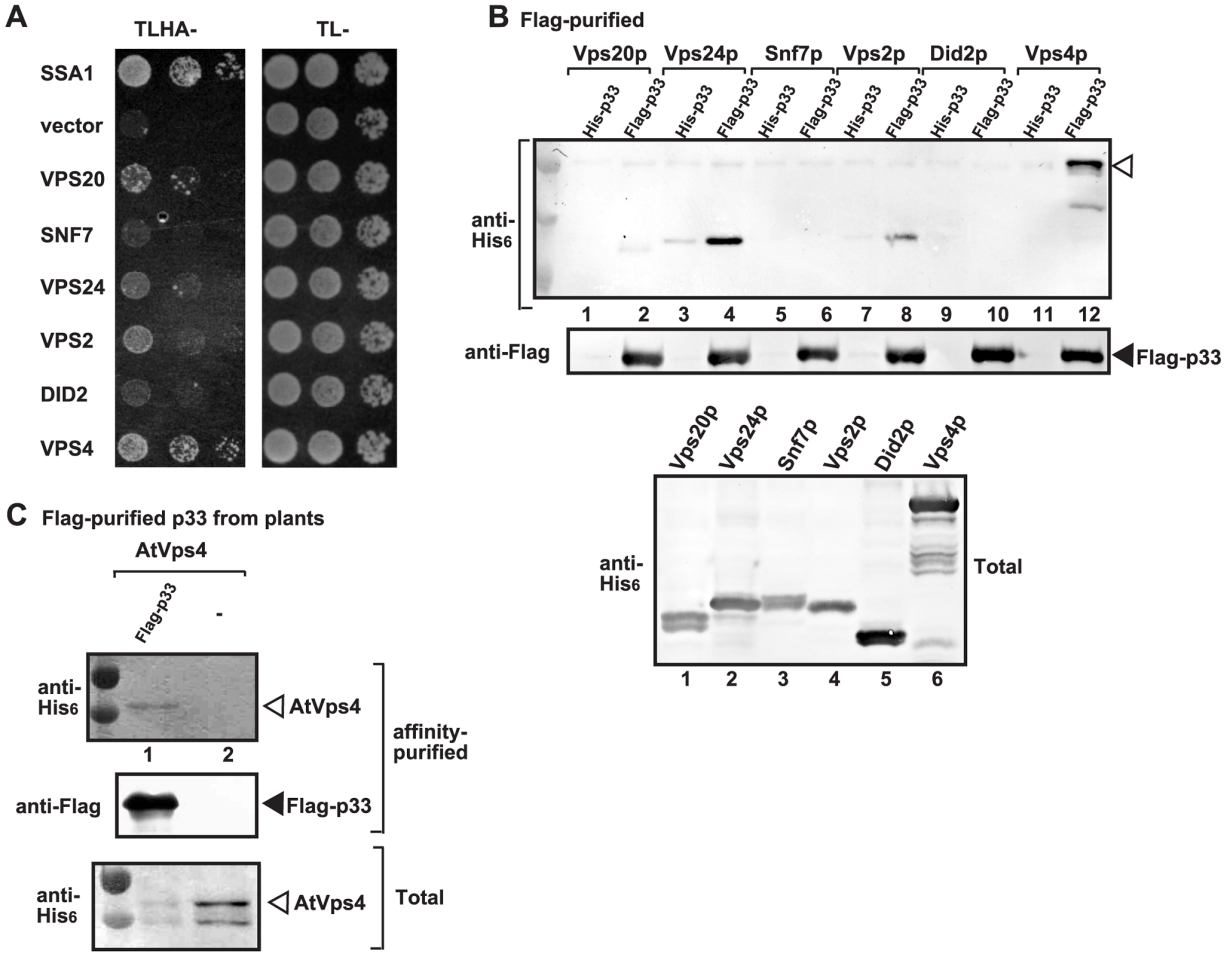
Interaction between p33 replication protein and Vps4p AAA+ ATPase and ESCRT-III proteins. (A) The split ubiquitin assay was used to test binding between p33 and Vps4p and the shown ESCRT-III proteins in yeast. The bait p33 was co-expressed with the shown prey proteins. *SSA1* (HSP70 chaperone), and the empty prey vector (NubG) were used as positive and negative controls, respectively. (B) Co-purification of Vps4p and the shown ESCRT-III proteins with the p33 replication protein. Top panel: Western blot analysis of co-purified His_6_-tagged cellular proteins with Flag-affinity purified p33 from solubilized membrane fraction from yeast cells. Vps4p and the ESCRT-III proteins were detected with anti-His antibody. The negative control was His_6_-tagged p33 purified from yeast extracts using a FLAG-affinity column. Middle panel: Western blot of purified Flag-p33 detected with anti-FLAG antibody. Bottom panel: Western blot of His_6_-tagged Vps4p and the ESCRT-III proteins in the total yeast extracts using anti-His antibody. (C) Co-purification of AtVps4 with the tombusvirus p33 replication protein. His_6_-AtVps4, p33-FLAG and p19 (suppressor of gene silencing) were co-expressed from pGD plasmids introduced by co-agro-infiltration of *N. benthamiana* leaves. Top panel: Western blot analysis of co-purified His_6_-tagged cellular AtVps4 with Flag-affinity purified FLAG-p33 (derived from the closely-related cucumber necrosis virus) from membrane fraction. AtVps4 was detected with anti-His antibody. The negative control was purified from yeast extracts using a FLAG-affinity column. Middle panel: Western blot of purified p33-FLAG detected with anti-FLAG antibody. Bottom panel: Western blot of His_6_-AtVps4 protein in the total plant leaf extracts using anti-His antibody. The left-most lanes (not numbered) in panel B–C are protein molecular weight markers.

The formation of spherule-like structures during TBSV replication on the peroxisomal membrane surfaces and the effect of various ESCRT proteins on tombusvirus replication [15], [48], suggest that some of the co-opted cellular ESCRT proteins might play noncanonical functions. To gain insights into the functions of the co-opted ESCRT proteins during tombusvirus replication, first we analyzed if p33 replication protein could interact with ESCRT-III components or the Vps4p AAA+ ATPase. The split-ubiquitin-based yeast two-hybrid assay (membrane-based MYTH assay) between the tombusvirus p33 and the yeast ESCRT-III components or Vps4p revealed strong interaction between p33 and Vps4p (Fig. 1A). This is surprising, since Vps4p is known to interact with the ESCRT-III components to recycle them from the endosome [59], [62], while recycling of the peroxisome-bound p33 to the cytosol is unlikely to happen and not yet documented. In addition, we observed good interactions between p33 and Vps2p, Vps20p, and Vps24p ESCRT-III factors (Fig. 1A). The most abundant ESCRT-III factor, namely Snf7p, whose deletion greatly affected TBSV replication [15], and Did2p interacted only weakly with p33 (Fig. 1A). Affinity-based co-purification experiments from the membrane fraction of yeast confirmed that Vps4p strongly interacted with p33 (Fig. 1B, lane 12), while the interaction of Vps2p and Vps20p with p33 was also detectable (Fig. 1B, lanes 2 and 8). Unlike in the MYTH assay, the co-purification experiments suggested strong interaction between p33 and Vps24p (Fig. 1B, lane 4). We could not co-purify Snf7p and Did2p with p33 (Fig. 1B, lanes 6 and 10). Altogether, Vps4p showed consistently the strongest interaction with the p33 replication protein and this interaction is unexpected and could play a direct role in TBSV replication.

To confirm that the interaction between the viral replication protein and Vps4p also occurs in plants, we co-expressed the *Arabidopsis thaliana* AtVps4 in *Nicotiana benthamiana* leaves together with the FLAG-tagged tombusvirus p33 replication protein (Fig. 1C). After isolation of the membrane-bound replicase from the leaves and solubilization of the membrane fraction with nonionic detergent, we FLAG-affinity purified p33, followed by Western blotting. This approach revealed co-purification of AtVps4 with the tombusvirus p33 (Fig. 1C, lane 1), while AtVps4 was missing after purification in the sample prepared from leaves lacking p33 (lane 2). Thus, similar to yeast, the tombusvirus p33 replication protein also interacts with AtVps4 in plant leaves, suggesting subversion of Vps4 for viral activities.

**Figure 2:**
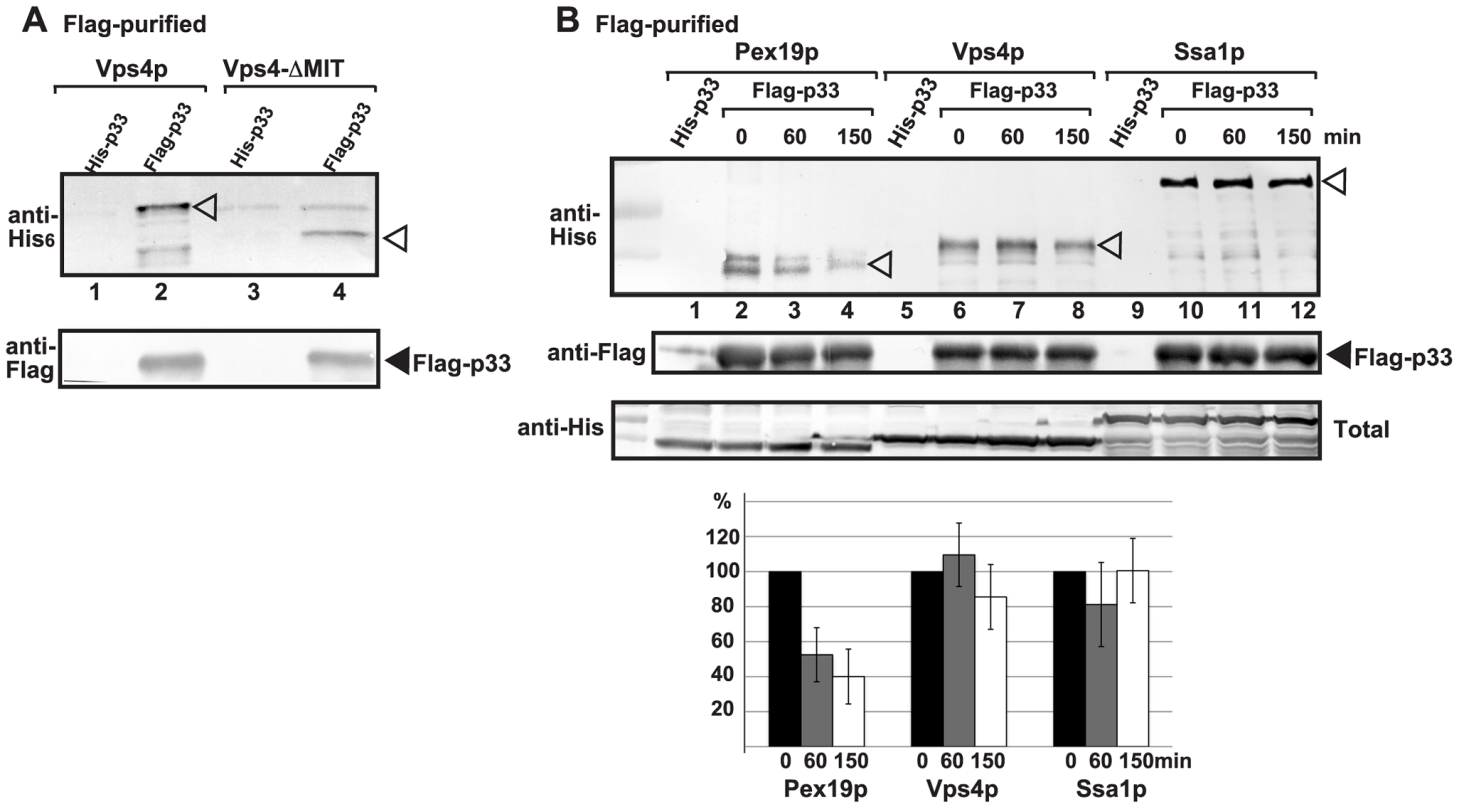
Vps4p is a permanent component in the tombusvirus VRCs. (A) The VRCs present in the membrane fraction of *vps4Δ* yeast expressing Flag- or His_6_-tagged p33, p92 and the DI-72 repRNA were solubilized with NP-40, followed by FLAG-affinity purification. Top panel: Western blot analysis of the co-purified His_6_-tagged Vps4p or its N-terminally truncated version (carrying the ATPase domain) in the purified replicase preparations with anti-His antibody. Bottom panel: Western blot analysis of Flag-tagged p33 with anti-Flag antibody. (B) Testing the presence of cellular factors in the VRCs after blocking cellular translation by cyclohexamide. Top panel: Western blot analysis shows the co-purified His_6_-tagged cellular proteins with the viral replicase isolated at the shown time points. See further details in panel A. Middle panel: Western blot analysis of the purified Flag-tagged p33 with anti-Flag antibody. Bottom panel: Western blot analysis of His_6_-tagged cellular proteins in the total yeast lysates with anti-His antibody. Note that “total” represents the total protein extract from yeast expressing the shown proteins. The left-most lanes (not numbered) are protein molecular weight markers. Each experiment was repeated three times.

Since Vps4p showed strong interaction with the tombusvirus p33 replication protein, we tested if Vps4p is part of the tombusvirus VRC. Interestingly, the affinity-purified tombusvirus replicase contained Vps4p (Fig. 2A, lane 2 versus 1). This finding highlights the possibility that Vps4p is a permanent component of the VRC (i.e., not rapidly recycled), such as Hsp70 [40]. To test this, we added cyclohexamide to yeast to prevent new p33 and p92^pol^ translation, thus formation of new VRCs. Then, we measured the level of Vps4p in the affinity-purified replicase at various time points to study if Vps4p is released from the VRCs. As a control, we used Ssa1p Hsp70, whose amount did not change within 150 min, confirming that Ssa1p remained stably associated with the existing tombusvirus VRCs (Fig. 2B, lanes 10–12). In contrast, the amount of Pex19p peroxisomal shuttle protein, which is involved in the delivery of p33 and p92^pol^ to the peroxisomes [64] before its getting recycled to the cytosol, decreased by 60% after 150 min of incubation in the presence of cyclohexamide (Fig. 2B, lanes 2–4). Interestingly, the amount of Vps4p did not change significantly in the affinity-purified replicase preparations during this time period (Fig. 2B, lanes 6–8), suggesting that Vps4p is likely a permanent component of the tombusvirus VRCs. Obviously, this is different from the canonical role of Vps4p, which is quickly recycled from the endosomal membranes to the cytosol after the disassembly of the endosome-bound ESCRT-III structures [59], [62]. Additionally, the N-terminally truncated Vps4p carrying the ATPase domain, but lacking the MIT domain, which is responsible for interaction with the ESCRT-III proteins, was recruited to the VRCs (Fig. 2A, lane 4), suggesting unique interaction between p33 and Vps4p.

### Binding of Vps4p to the p33 replication protein involves both the MIT and ATPase domains

**Figure 3:**
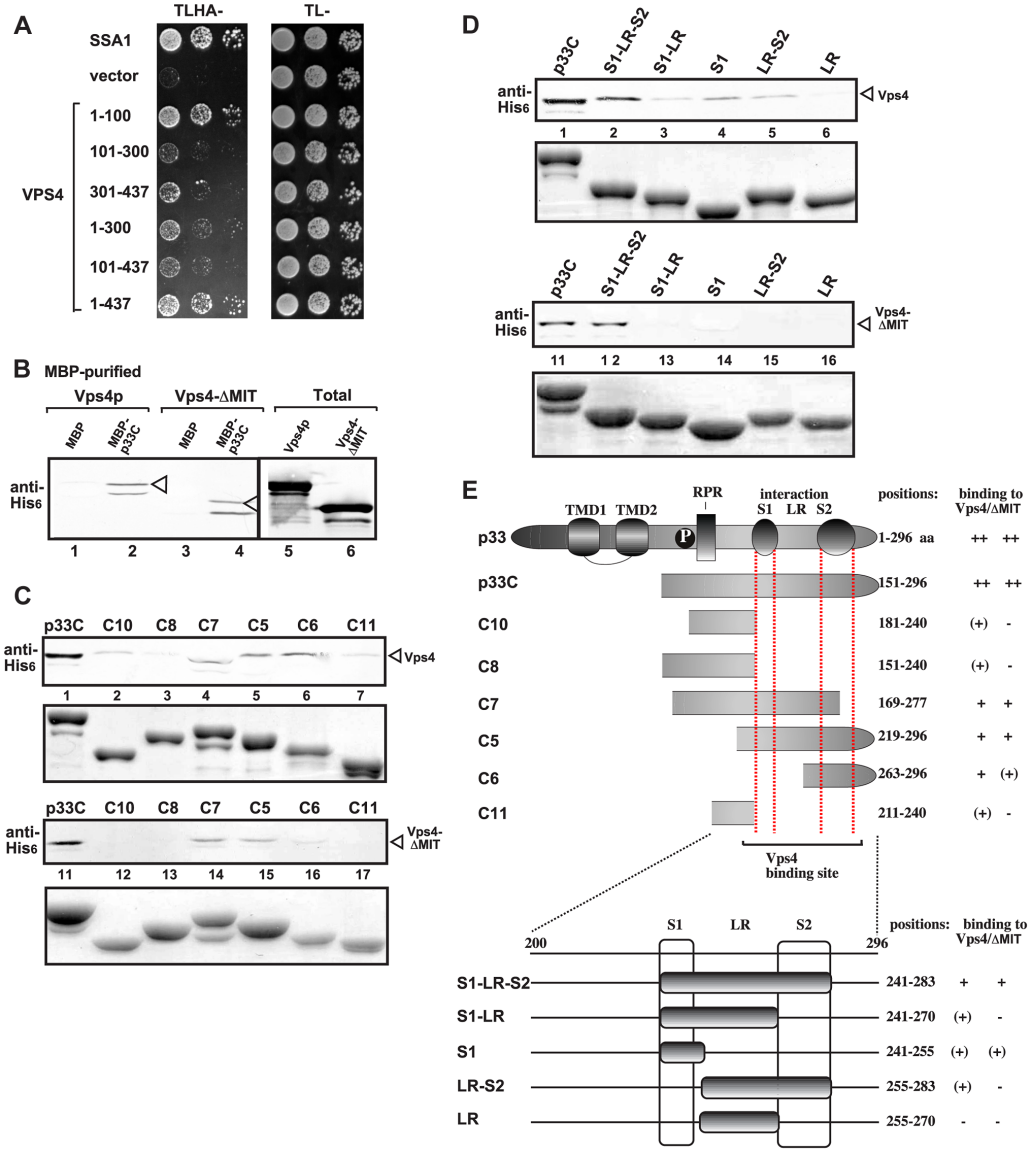
Interaction between Vps4p and the tombusvirus p33 replication protein. (A) Split ubiquitin MYTH assay was used to test binding between p33 and the full-length yeast Vps4p (construct 1–437) or its truncated derivatives. The bait p33 was co-expressed with the prey Vps4p protein in yeast. SSA1 (HSP70 chaperone), and the empty prey vector (NubG) were used as positive and negative controls, respectively. (B) Affinity binding assay to detect interaction between His_6_-tagged Vps4p and the MBP-tagged viral p33 protein (the C-terminal portion). The MBP-tagged viral protein or MBP control produced in *E. coli* was immobilized on amylose-affinity columns. Then, His_6_-tagged Vps4p or its ATPase domain expressed in *E. coli* was passed through the amylose-affinity columns with immobilized MBP-tagged proteins. The affinity-bound proteins were eluted with maltose from the columns. The eluted proteins were analyzed by Western blotting with anti-His antibody to detect the amount of His_6_-tagged Vps4p specifically bound to MBP-tagged viral protein. (C–D) Affinity binding assay to map the interaction sites in p33 with His_6_-tagged Vps4p. The MBP-tagged viral proteins produced in *E. coli* were immobilized on amylose-affinity columns. Then, His_6_-tagged Vps4p expressed in *E. coli* was passed through the amylose-affinity columns with immobilized MBP-tagged proteins. See further details in panel B. The lower panels show the Coomassie blue-stained SDS-PAGE of the eluted MBP-tagged p33 derivatives. (E) Summary of the Vps4p binding region in p33. Schematic representation of the TBSV p33 and its truncated derivatives used in the binding assay. The various domains include: TMD, transmembrane domain; RPR, arginine-proline-rich RNA binding domain; P; phosphorylated serine and threonine; S1 and S2 subdomains involved in p33:p33/p92 interaction, LR is a linker region.

To map the binding sites in Vps4p, we used the split ubiquitin assay that revealed that the N-terminal MIT domain, which binds to the ESCRT-III proteins [59], [62], bound efficiently to the full-length p33 (construct 1–100 versus the full-length Vps4 construct 1–437, Fig. 3A). We also observed weaker, but substantial binding between the C-terminal ATPase domain and p33 (Fig. 3A). We confirmed the interaction using the C-terminal ATPase domain and compared with the full-length Vps4p in a pull down assay with p33 (Vps4-ΔMIT, Fig. 3B). The interaction between the ATPase domain and p33 is not abolished by addition of ATP (not shown). Overall, the binding of p33 to Vps4p is different from the binding between Vps4p and the cellular ESCRT-III components that target only the MIT domain and leads to the Vps4p-driven recycling of the ESCRT-III components from the membranes back to the cytosol. We suggest that the unique interaction between the p33 and Vps4p subverts Vps4p for viral replication, leading to association of Vps4p with the membrane-bound replicase, and likely altering the canonical function of Vps4p.

Detailed mapping of p33 sites interacting with the Vps4p or the ATPase domain revealed that the very C-terminus of p33, which contains the p33:p33/p92 interaction sites, is involved in binding to Vps4p (Fig. 3C–F). Binding of the ATPase domain of Vps4p to p33 mostly overlaps with that of the full-length Vps4p in this assay. Altogether, the binding between Vps4p and p33 involves unique interactions that will require high-resolution structural studies.

### Vps4p co-localizes with the viral double-stranded RNA in yeast cells replicating TBSV RNA

**Figure 4:**
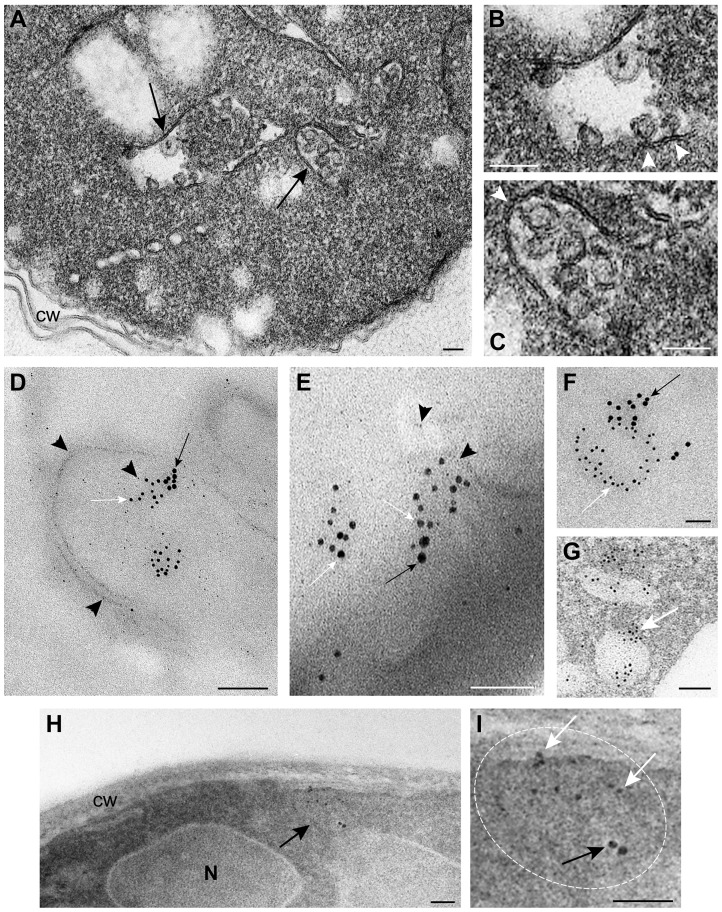
TBSV VRCs assemble in yeast intracellular membranes that contain p33, viral dsRNA and the ESCRT protein Vps4p. (A) TEM of a stained ultra-thin section of a yeast cell (BY4741) replicating TBSV repRNA. Characteristic spherule-like vesicles are assembled in a peripheral membranous compartment (arrows). Enlargements of the structures marked with arrows in panel A are shown in (B) and (C). Arrowheads point to contacts (necks) between the spherules and the membrane of the compartment. The structure in panel B exhibits a rather empty center while that in panel C is filled with membranes. (D) and (E) VRCs in yeast peripheral structures as studied by immunogold and METTEM. Ultra-thin sections of yeasts expressing HA-tagged Vps4p (detected with anti-HA and 10 nm gold particles, black arrows in panels D, E and F), replicating TBSV repRNA (detected with anti-dsRNA antibodies and 5 nm gold particles, white arrows in panels D, E and F) and MT-tagged p33 (∼1 nm gold nano-clusters associated to MT, black arrowheads in panels D and E); sections were studied without previous staining. P33, dsRNA and Vps4p colocalize in the same peripheral compartments. (G) to (I) Ultra-thin sections of yeast cells from the same sample after staining with uranyl acetate and lead citrate. MT-p33 nanoclusters are no longer visible but cell compartments are better distinguished. Vps4p and dsRNA are clustered together in peripheral structures compatible with the spherule-containing compartment shown in panel C. (G) White arrow points to 5 nm colloidal gold particles bound to anti-dsRNA antibodies. (I) Enlargement of the membranous compartment marked with an arrow in panel H. Immunogold labeling shows co-localization of dsRNA (white arrows) and Vps4p (black arrow). Bars, 50 nm in panels A, B, C, E, F, G, H and panel I; 100 nm, in D.

We have developed an EM-based assay to visualize the tombusvirus-induced spherule-like structures in yeast, which are known to form in infected plant tissues [15], [48]. EM images revealed the presence of single-membrane vesicle-like structures of ∼25–50 nm (Fig. 4A–C) that were missing in wt yeast not expressing the tombusvirus replication proteins (Fig. S1A–B). These vesicles likely represent the spherules seen in TBSV-infected plant tissues [15].

To show if Vps4p is localized to the tombusvirus VRCs, we used immuno-gold EM of yeast cells co-expressing HA-tagged Vps4p, and MT (Metal-binding protein metallothionein)-tagged p33 that was visualized by Metal-Tagging Transmission Electron Microscopy (METTEM) [65] and replicating the TBSV repRNA, which was detected through using a dsRNA-specific antibody [65] (Fig. 4D–G). These samples were processed in the absence of osmium tetroxide that would destroy most protein epitopes and would mask the 1 nm nano-clusters associated to p33-MT. We frequently observed Vps4p in the close vicinity of the dsRNA (present in the VRCs) based on using different sized gold particles for immuno-gold EM. It was difficult to detect Vps4p in the areas of the yeast cells lacking dsRNA (Fig. S2). We suggest that Vps4p is likely concentrated in the VRCs due to binding to p33, thus facilitating detection of Vps4p in yeast membranous compartments replicating TBSV.

For an adequate visualization of gold nanoclusters bound to p33-MT, cells in Fig. 4D and 4E were processed in the absence of osmium tetroxide and contrasting agents as described. Under these conditions intracellular membranes are invisible; however, some membranes can be visualized if these ultra-thin sections are stained with uranyl acetate and lead citrate (Fig. 4G–I) (see Materials and Methods). Stained cells showed that Vps4p and the viral dsRNA were surrounded by membranes (Fig. 4G–I), supporting the model that Vps4p is part of the functional membrane-bound tombusvirus VRCs.

**Figure 5:**
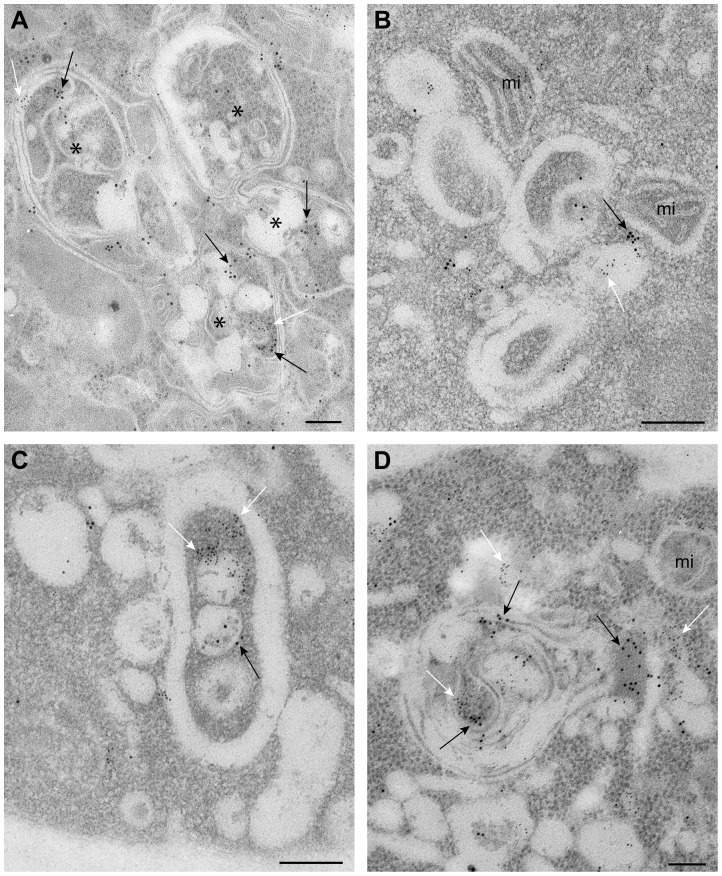
Complex membranous compartments containing both tombusvirus VRCs and the ESCRT protein Vps4p fused with MT and HA tags in yeast. (A) General organization of the complex membranous compartments containing the tombusvirus VRCs as seen in ultrathin sections of yeast cells (BY4741) processed by immunogold labeling and stained with uranyl acetate and lead citrate. Vps4p-MT-HA was detected with anti-HA and 10 nm gold particles (black arrows) and replicating TBSV repRNA with anti-dsRNA antibodies and 5 nm gold particles (white arrows). Asterisks mark the center of spherule-containing membranous structures that are connected to form a complex compartment where Vps4p and dsRNA are clustered together. (B) Membranous structures containing spherules and VRCs are surrounded by mitochondria (mi). Note that proximal location of mitochondria might be useful for robust virus replication that is dependent on ATP generation. This arrangement of VRCs and mitochondria are frequently observed in other viral replication platforms. (C–D) Membranous structures with spherules of ∼70 nm containing both Vps4 and dsRNA. The Vps4p-MT-HA and dsRNA containing areas are connected with a complex system of membranes. Bars, 100 nm.

To enhance the visualization of Vps4p, we over-expressed His_6_-MT-tagged Vps4p in yeast cells replicating TBSV repRNA. This approach facilitated the frequent observation of Vps4p in the close vicinity of the TBSV dsRNA by immuno-gold EM (Fig. 5). Interestingly, the spherule-containing membranous structures are frequently connected to form a complex compartment where Vps4p and dsRNA are clustered together (Fig. 5). Altogether, the co-localization (proximal location based on immuno-EM) of Vps4p and the tombusviral dsRNA in yeast cells suggests that Vps4p is recruited to the VRCs actively involved in TBSV RNA replication.

### The viral (+)RNA co-purifies with Vps4p from yeast cells replicating TBSV RNA

**Figure 6:**
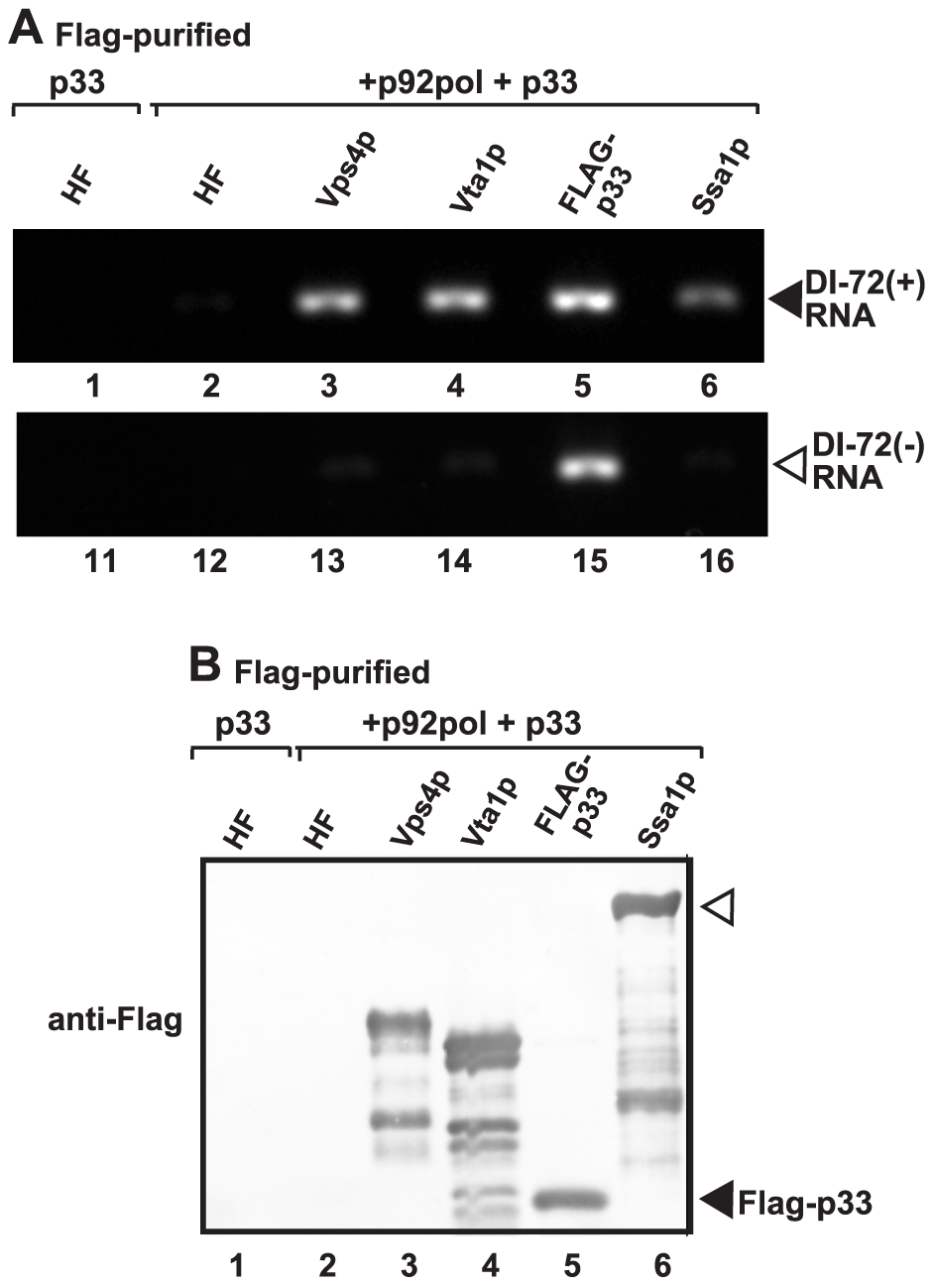
Co-purification of the viral (+)RNA with Vps4p. (A) The viral replicases present in the membrane fraction of yeast expressing Flag-tagged Vps4p and His_6_-tagged p33, p92 and the DI-72 repRNA were solubilized with Triton-X100, followed by FLAG-affinity purification. The agarose gel image shows the RT-PCR analysis of the co-purified DI-72(+) top panel or DI-72(−) repRNA (bottom panel). Similar approach was used to detect the viral RNA with RT-PCR in samples obtained via FLAG-affinity purification through Flag-tagged Vta1p accessory factor, Flag-p33 replication protein and Flag-Ssa1p Hsp70 host factor. A His_6_-Flag fusion protein served as a negative control. (B) Western blot analysis of the Flag-affinity purified proteins from the samples shown in panel A using anti-Flag antibody.

If Vps4p is part of the tombusvirus VRCs, it is likely associated with the neck structure of tombusvirus-induced spherules, which is predicted to serve as exit places for the newly synthesized (+)RNA from the VRCs. Therefore, we tested if Vps4p is in contact with the tombusviral RNA. Affinity purification of Vps4p from the membrane fraction of yeast containing the tombusvirus VRCs resulted in co-purification of the TBSV (+)repRNA (Fig. 6A, lane 3). Interestingly, affinity purification of Vta1p accessory protein, which facilitates the formation of the functional Vps4p rings with ATPase function from the inactive Vps4p dimers [62], also resulted in co-purification of the TBSV (+)repRNA (Fig. 6A, lane 4). As a positive control, we also used the tombusvirus p33 replication protein (purified via FLAG-tag), which also resulted in co-purification of the TBSV (+)repRNA (Fig. 6A, lane 5). The Ssa1p Hsp70 chaperone, which is another permanent component of the tombusvirus VRCs also resulted in co-purified (+)repRNA, although in a lesser amount than p33 or Vps4p (Fig. 6A, lane 6 versus 3 and 5). The negative control (HF, a peptide sequence containing His_6_-FLAG sequence expressed from the empty expression plasmid) did not contain any detectable TBSV (+)repRNA, excluding that the viral RNA bound nonspecifically to the affinity resin or the FLAG-antibody.

We also tested the presence of (−)repRNA (which might be present in a dsRNA form within the VRCs) in the above samples. As expected, the p33 replication protein preparation contained (−)repRNA (Fig. 6A, lane 15), while Vps4p, Vta1p, Ssa1p and the negative control samples did not contain (−)repRNA (Fig. 6A). Based on these data, we suggest that the Vps4p/Vta1p complex might be in direct contact with the TBSV (+)repRNA. This contact is unlikely via p33 replication protein since both (+) and (−)repRNAs were co-purified with p33.

### Vps4p is involved in the formation of nuclease-resistant tombusvirus replicase *in vitro*

**Figure 7:**
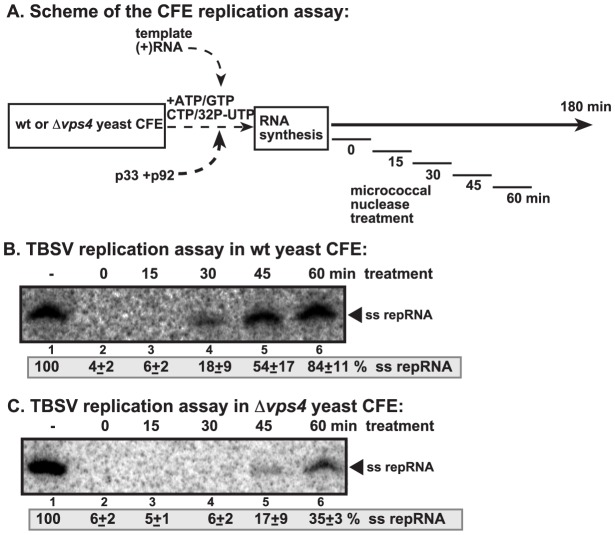
Increased nuclease-sensitivity of the tombusvirus replicase assembled in yeast with deletion of *VPS4* ESCRT gene. (A) Scheme of the CFE-based TBSV replication assay. CFEs prepared from wt and *vps4Δ* yeasts were programmed with DI-72(+) repRNA and purified recombinant p33 and p92^pol^ replication proteins. Micrococcal nuclease was added to the CFE for 15 minutes at various time points as shown (after which the nuclease was inactivated by EGTA). The total length of the CFE-based replication assay was 3 h for each sample. (B–C) Denaturing PAGE analysis of *in vitro* replicase activity of the CFEs from wt and *vps4Δ* yeasts. The untreated preparation was chosen as 100%. Note that the repRNA could be protected from micrococcal nuclease degradation by the membrane-associated viral replicase complex if formed correctly.

To obtain evidence that Vps4p is involved in the formation of tombusvirus VRCs, we took advantage of a cell-free TBSV replication assay based on TBSV-free yeast cell-free extracts (CFE) prepared from wt or *vps4Δ* yeast [41]. The CFE supports the assembly of the VRCs when purified recombinant p33 and p92 and (+)repRNA are added. Micrococcal nuclease was also added for 15 min (after which it was inactivated) to the assay at various time points to test the nuclease-sensitivity of the viral repRNA within newly assembled membrane-bound VRCs (Fig. 7A). The CFE from wt yeast was able to assemble nuclease-insensitive VRCs in ∼45–60 min (Fig. 7B, lane 5). In contrast, the VRCs assembled with CFE from *vps4Δ* yeast produced only small amounts of repRNA in the presence of nuclease, suggesting that Vps4p is required for the assembly of the nuclease-insensitive tombusvirus VRCs *in vitro*. The nuclease-insensitivity of the viral RNA requires cellular membranes and ATP/GTP-dependent VRC assembly and is not due to protein coating of the viral RNA, as shown previously [40], [41].

### Vps4p is required for the formation of spherule-like structures induced by tombusvirus replication proteins in yeast

**Figure 8:**
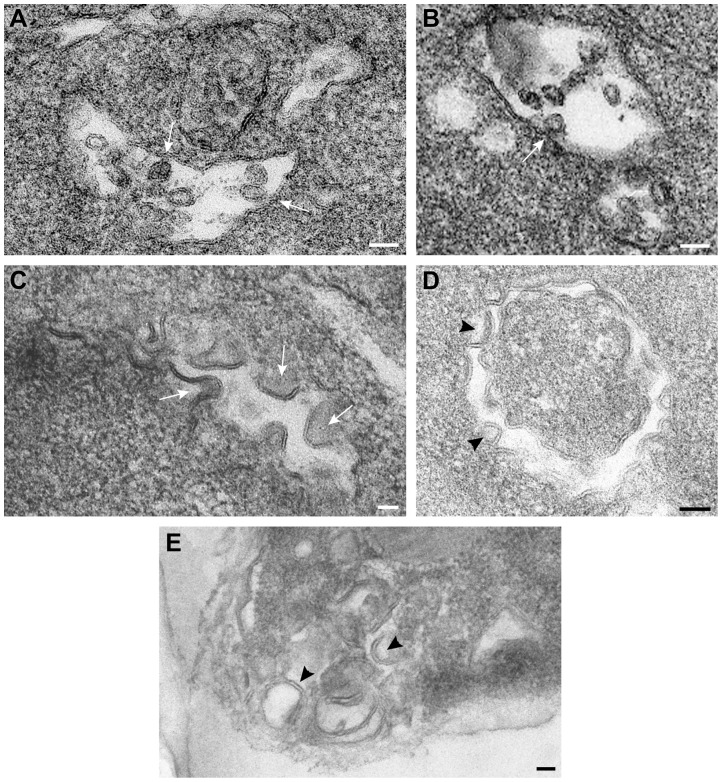
Vps4p is required for the formation of spherule-like structures induced by tombusvirus replication proteins in yeast. TEM of stained ultra-thin sections of yeast cells replicating TBSV repRNA and processed for ultrastructural analysis. (A) and (B) Wild type yeast cells with characteristic membranous compartments with tombusvirus-induced spherules. Arrows point to connections (neck-like constrictions) between spherules and the membrane of the intracellular compartment. (C) and (D) Peripheral membranous compartments in *vps4Δ* yeasts. Crescent-shaped membranes representing incompletely formed VRCs with wide openings face the lumen of the compartment. (E) Peripheral membranous compartments in *vps24Δ* yeasts. Crescent-shaped membrane structures face the lumen of the compartment but apparently fail to complete the spherule constriction since they have wide openings to the cytosol (white arrows in panel C, black arrowheads in panels D and E). Bars, 50 nm.

To visualize the tombusvirus VRCs in yeast lacking Vps4p, we performed EM imaging of *vps4Δ* yeast replicating TBSV repRNA. Interestingly, unlike the characteristic tombusvirus-induced spherule-like structures in wt yeast (Fig. 8A and B), the membranes from *vps4Δ* yeast expressing the p33 and p92 replication proteins and the repRNA showed different deformations (Fig. 8C and D). These crescent-shaped structures likely represent incompletely formed spherules with wide openings containing viral replicases. Similar structures (either spherules or open crescent-shaped structures) were not visible in control yeasts not expressing the viral proteins (not shown). Similar crescent-shaped membrane-structures were also visualized by EM in *vps24Δ* yeast replicating TBSV repRNA (Fig. 8E), suggesting that ESCRT-III components are also likely needed for proper deformation of membranes and VRCs formation. We interpret these data that the tombusvirus replication proteins could not induce the formation of complete spherule-like structures in *vps4Δ* or *vps24Δ* yeasts.

**Figure 9:**
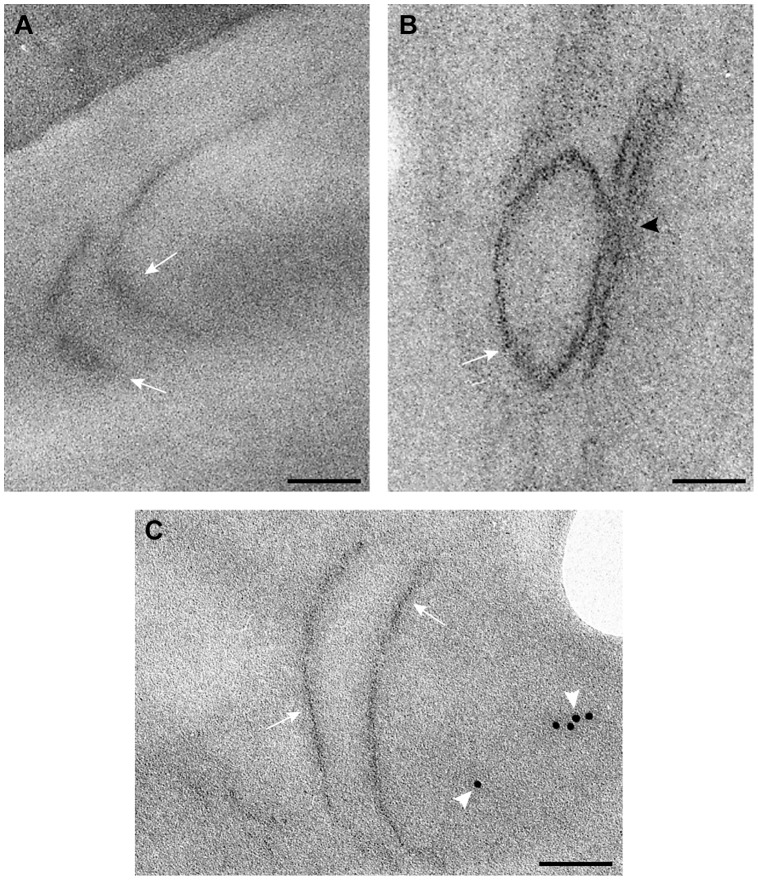
Detection of MT-tagged p33 replication protein in wild type and *vps4Δ* yeast. A) Electron-dense small nano-clusters associated to MT-tagged p33 reveal the presence of p33 protein molecules in elongated membrane-like structures (white arrows) of the periphery of *vps4Δ* yeast. B) In wt yeast MT-tagged p33 molecules (white arrow) concentrate in vesicle-like structures associated to peripheral membranous compartments. The black arrowhead points to the connection between the spherule and the compartment membrane. C) Immunogold labeling with anti-dsRNA antibodies and a 10 nm colloidal gold conjugate in ultra-thin sections of *vps4Δ* yeast. Weak signals associated to viral dsRNA (white arrowheads) are detected in peripheral membranes where MT-tagged p33 molecules are present. Bars, 50 nm.

To visualize the tombusvirus p33 replication protein in yeast subcelluar membranes, we used Metal-Tagging Transmission Electron Microscopy (METTEM) [66] with yeast expressing MT-tagged p33 replication protein. The MT-p33 molecules were present in elongated structures in *vps4Δ* yeast (Fig. 9A), while they could form round vesicle-like structures in wt yeast (Fig. 9B). These data are consistent with the model that Vps4p is required for the formation of tombusvirus-induced spherule-like structures in yeast cells. Labeling with anti-dsRNA antibodies revealed weak to moderate signals in *vps4Δ* yeast (Fig. 9C). These results suggest that, in the absence of Vps4p, the abnormal VRCs are still able to support some repRNA replication. However, the level of repRNA replication is low as shown in the CFE-based replication assay (∼75% less repRNA replication in this strain than in wt) [15]. Thus, replication of repRNA is inefficient in *vps4Δ* yeast and the repRNAs is less protected in wide open VRCs and much more sensitive to degradation.

## Discussion

**Figure 10:**
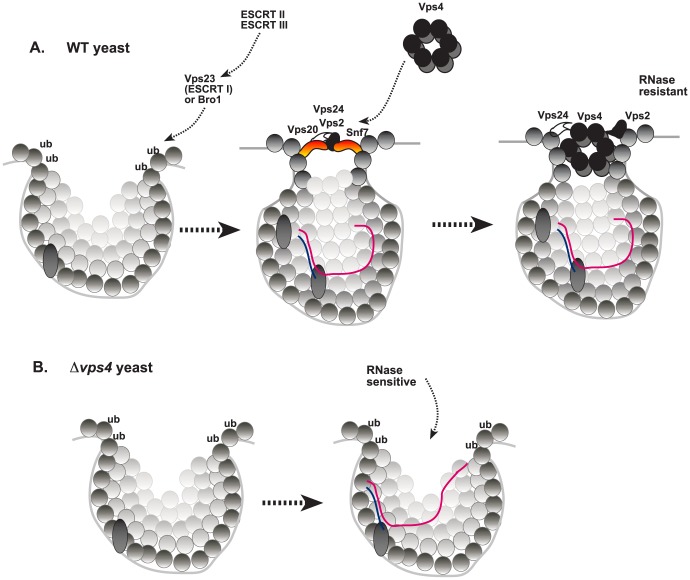
A model on the role of Vps4p during TBSV replication in wild type yeast and the replication defect in *vps4Δ* yeast. (A) The formation of tombusvirus-induced spherules (membrane invaginations) in the peroxisomal membranes is facilitated by the recruitment of the shown ESCRT proteins by the monoubiquitinated p33 replication protein. While the ESCRT-III components are likely recruited to the sites of replication to facilitate VRC assembly, the role of ESCRT-II factors, if any, are currently unknown. The TBSV replication model predicts that the scission of the membranous neck and the removal of the ESCRT-III proteins (Vps20p, Vps2p, Vps24p and Snf7p) by Vps4p AAA+ ATPase might be blocked by interaction with the p33 replication protein. Altogether, Vps4p becomes a permanent component of the spherule-like structure. Vps4p might also be involved in the release of the newly-made TBSV (+)RNA (not shown). (B) In the absence of Vps4p, the ESCRT-III proteins do not work properly and tombusvirus replication proteins could only form membrane invaginations with large openings that make the viral replicase complex RNase-sensitive.

Co-opted host proteins, such as Hsp70 and eEF1A, play important roles in the assembly of the tombusvirus VRCs *in vitro*, in yeast and plant cells [30], [46], [63]. We have also shown previously that TBSV recruits the cellular ESCRT proteins via binding to Vps23p ESCRT-I protein and Bro1p ESCRT-accessory protein, likely leading to subversion of the cellular ESCRT machinery consisting of additional ESCRT-I and ESCRT-III components and Vps4p AAA+ ATPase [15], [63]. Accordingly, deletion of not only *VPS23* or *BRO1*, but several other ESCRT genes in yeast or over-expression of dominant-negative mutants in plant leaves led to 10-to-20-fold reduction in TBSV RNA accumulation [15]. Therefore, a model was proposed that the cellular ESCRT proteins are involved in membrane bending/invagination and viral spherule formation during the assembly of the membrane-bound tombusvirus replicase complexes. However, the recruitment of the ESCRT machinery to the membrane is expected to lead to pinching off the vesicle (scission of the membrane and closure of the neck structure) into the lumen of the organelle as shown during the virion budding of HIV or MVB formation in the cell [57]–[59]. Formation of closed vesicles inside the peroxisomes (if pinching off the spherule would take place, sending the newly formed vesicle into the organellar lumen) should be a problem for tombusviruses. This is because the closed replication vesicles inside the peroxisomes would be deprived from accessing ribonucleotides needed for robust viral RNA synthesis from the cytosol and also face difficulties to release the newly made (+)RNA progeny back to the cytosol for additional viral processes, such as new rounds of translation and replication, cell-to-cell movement and encapsidation. Therefore, it is more plausible that tombusviruses halt the membrane budding process into the organellar lumen and stabilize the neck structure as schematically shown in Fig. 10A. Accordingly, the spherule-like structures could be visualized in tombusvirus infected plant tissues [15], [48] or in yeast (this work). These spherules could have controlled communication with the cytosol [19].

But how the neck of the spherules could be stabilized to prevent the formation of luminar vesicles containing trapped VRCs inside the vesicles? Based on the results from this work, we propose that tombusviruses recruit Vps4p and incorporate it as a permanent component of the VRCs. The supporting data include: (i) the strong binding between the p33 replication protein and Vps4p (Fig. 1); (ii) co-purification of p33 and Vps4p from yeast membrane fraction containing the VRCs (Fig. 1B–2A) or from plant leaves co-expressing the homologous AtVps4 and the tombusvirus p33 (Fig. 1C); and (iii) the extended presence of Vps4p, similar to Ssa1p Hsp70 protein, in the VRCs when new VRC formation is blocked (Fig. 2B). This is unlike the cellular Pex19p shuttle protein, which is recycled from the VRCs back to the cytosol (Fig. 2B). This is an unexpected feature of Vps4p, which should be easily recycled from the membranes after the disassembly of the membrane-bound ESCRT-III structures based on its canonical function [59]. (iv) Co-purification of the viral (+)repRNA with Vps4p from the membrane-bound VRCs (Fig. 6), suggesting that Vps4p might be in contact with the viral RNA. All the above features predict noncanonical functions of Vps4p, which has a role in disassembling and recycling membrane-bound ESCRT-III structures to the cytosol as ESCRT-III protein monomers [57]–[59], [62].

We also found Vps4p in the close proximity of the viral dsRNA using immuno-gold EM (Figs. 4–5). The production of dsRNA likely occurs after the assembly of the VRCs, thus Vps4p is predicted to remain a component of the assembled VRCs. Interestingly, we also observed association of Vps4p with the TBSV(+)repRNA from the membrane fraction of yeast (Fig. 6). Based on these data, we propose that one of the roles of Vps4p could be the stabilization of the neck structure of the spherule preventing scission of the membranes. Vps4p might have additional function(s), such as a role in selective release of the viral (+)repRNA progeny from the replicase during replication using the AAA+ ATPase activity of Vps4p. Additional, high resolution images will be needed to demonstrate this intriguing possibility. We also show the association of Vta1p accessory protein, which facilitates the formation of the functional Vps4p rings with ATPase function from the inactive Vps4p dimers [62], with the viral (+)repRNA (Fig. 6). Although further experiments will be needed to address the role of Vta1p during tombusvirus replication, the intriguing possibility is that Vps4p and Vta1p are part of the tombusvirus replicase and have functional roles.

In addition to Vps4p, some ESCRT-III proteins could also be involved in the putative stabilization of the neck structure, since we find interaction between p33 replication protein and several ESCRT-III factors, such as Vps24p, Vps2p and Vps20p (Fig. 1B). The interactions between p33 and the selected ESCRT-III components could prevent the membrane scission function of ESCRT-III, especially if Vps4p is also recruited by TBSV for noncanonical activities as suggested above. Accordingly, incomplete, crescent-like membrane structures were observed in *vps24Δ* yeast (lacking the critical Vps24p ESCRT-III component), suggesting the absence of neck-like structures in these membrane deformations. However, it is possible that the ESCRT-III (and ESCRT-I) components are co-opted by tombusviruses for assisting membrane-bending around the replicase, which should occur prior to the neck formation/stabilization.

The highly conserved Vps4p likely plays a similar role in tombusvirus replication in plants. Accordingly, we co-purified AtVps4 with the tombusvirus p33 replication protein from membrane fractions from *Nicotiana benthamiana* leaves (Fig. 1C). Moreover, we have previously found that expression of a dominant-negative mutant of AtVps4 in *N. benthamiana* leaves blocked tombusvirus replication and the formation of characteristic spherules [15]. Thus, the emerging picture is that the role of Vps4p and the ESCRT machinery is to aid building membrane-bound VRCs, which are nuclease-insensitive to avoid the recognition by the host antiviral surveillance system and the destruction of the viral RNA. It is likely that other (+)RNA viruses of plants and animals could subvert Vps4p and the ESCRT machinery for formation of VRCs, which require membrane deformation and spherule formation.

### Summary

In this paper, we document a novel, noncanonical role for the Vps4p AAA+ ATPase during TBSV replication. We propose that tombusviruses not only recruit the cellular ESCRT machinery for assembling the membrane-bound VRCs, but the virus could alter the activities of Vps4p and the ESCRT-III proteins to create new functional structures (spherules acting as VRCs) and possibly new activities. The recruitment of Vps4p and additional ESCRT proteins are needed for the assembly of the replicase complex, which could help the virus evade recognition by the host defense surveillance system and/or prevent viral RNA destruction by the gene silencing machinery.

## Materials and Methods

### Co-purification of FLAG-p33 and host proteins

To study co-purification of host ESCRT proteins with p33 replication protein, the yeast strain BY4741 was transformed with plasmids pGBK-FLAGp33-CUP1 (or pGBK-His33-CUP1 as control, see plasmids used in the Supplementary material) plus the pYES plasmids expressing 6×His-tagged ESCRT proteins from the GAL1 promoter (see supplementary Material and Methods S1 for additional information). The transformed yeasts were pre-grown in SD minimal media plus 2% glucose for 20 h at 29°C, then transferred to SD minimal media plus 2% galactose for 16 h at 29°C to induce over-expression of ESCRT proteins from the *GAL1* promoter. Then, the cultures were supplemented with 50 µM CuSO_4_ to induce expression of FLAG-p33 or His_6_-p33 from the *CUP1* promoter. The yeasts were collected by centrifugation after 8 h, washed in phosphate buffer saline (PBS) and then incubated in PBS plus 1% formaldehyde for 1 h on ice to cross-link proteins. Afterwards, the formaldehyde was quenched by adding glycine to a final concentration of 0.1 M. Then, yeasts were washed in PBS and processed to purify the FLAG-tagged p33 protein as described [31]. The FLAG purified fractions were eluted from the FLAG M2-agarose columns with SDS-PAGE loading buffer (without 2-mercaptoethanol). Then the eluted fractions were supplemented with 2-mercaptoethanol (5%) and boiled for 30 minutes to reverse cross-linking.

To study co-purification of His_6_-Vps4p and His_6_-Vps4_101–437_, the yeast strain BY4741 was transformed with plasmids pGBK-FLAGp33-CUP1/DI72-GAL1 (or pGBK-Hisp33-CUP1/DI72-GAL1 as control), pGAD-His92-CUP1 [67] and pYC-NT-VPS4 or pYC-NT-vps4_101–437_. Transformed yeasts were pre-grown in SD minimal media plus 2% glucose for 20 h at 29°C, then transferred to SD minimal media plus 2% galactose and 50 µM CuSO_4_ for 24 h at 29°C. The yeasts are then treated with 1% formaldehyde as above and processed for FLAG-p33 protein purification.

To analyze the time dynamics of host proteins association with p33, BY4741 was transformed with plasmids pGBK-FLAGp33-CUP1/DI72-GAL1 (or pGBK-Hisp33-CUP1/DI72-GAL1 as control), pGAD-His92-CUP1 [67] and either pYES-PEX19, pYES-VPS4 or pYES-SSA1. Transformed yeasts were pre-grown in SD minimal media with 2% glucose for 20 h at 29°C and then transferred to media with 2% galactose for 24 h at 29°C. The cultures were supplemented with 50 µM CuSO_4_ for 2.5 h to induce expression of the viral proteins. Then cycloheximide was added (100 µg/ml) to stop protein translation and samples were taken immediately (time 0), 60 and 150 minutes afterwards. Yeast cultures were treated with formaldehyde and processed for FLAG-p33 purification as above.

Purified FLAG-p33 was detected by western blot using anti-FLAG antibody followed by anti-mouse antibody conjugated to alkaline phosphatase. Co-purified His_6_-tagged host proteins were detected with anti-His antibody followed by anti-mouse antibody conjugated to alkaline phosphatase. Detection was performed with NBT and BCIP.

### Analysis of *in vitro* protein interactions

The pMAL c2x-derived plasmids described in the Supplementary materials were transformed into Epicurion Bl21-codon-plus (DE3)-R1L cells (Stratagene). Expression of MBP-tagged proteins was induced by IPTG as described [68]. pGEX-His-VPS4 and pGEX-His-vps4_101–437_ were also transformed into Epicurion Bl21-codon-plus (DE3)-R1L cells. Expression of GST-His_6_-tagged Vps4p and Vps4_101–437_ was essentially done as described [68] with the exception that cultures were incubated at 23°C during IPTG treatment. The cells were broken by sonication as described [68]. The lysates were incubated with GST resin (Novagen) for 1 h at 4°C. The GST resin was washed four times with column buffer [68] and then incubated for 3 h at 21°C with column buffer plus 1 mM 2-mercaptoethanol + 1 mM CaCl_2_ + 1 U Thrombine (Novagen) to cleave the His_6_-Vps4p and His_6_-Vps4_101–437_ from the column-bound GST protein. The amylose columns containing the MBP or MBP-tagged p33 portions were then incubated with 15 µg of the purified His_6_-Vps4p or His_6_-Vps4_101–437_ for 1 h at 4°C. The columns were then washed five times with column buffer and the MBP-tagged proteins were eluted with column buffer plus 10 mM maltose. The MBP-tagged proteins were analyzed by SDS-PAGE electrophoresis followed by coomassie staining. Bound His_6_-Vps4p or His_6_-Vps4_101–437_ were detected with anti-His antibody followed by alkaline phosphatase- conjugated anti-mouse and NBT/BCIP.

### Analysis of *in vivo* protein interactions by yeast membrane two-hybrid assay

The yeast membrane two-hybrid assay, based on the split-ubiquitin system (Dualsystems) has been described before [31]. The plasmid pGAD-BT2-N-His33 was co-transformed with pPR-N-RE derived constructs into the reporter yeast strain NMY51. Transformed yeasts colonies were suspended in 100 µl of water and serially diluted (10-fold) in water. 6 µl of each dilution were spotted onto TLHA- plates, to score for interaction, or TL- plates, as growth controls.

### Co-purification of host proteins and viral RNA

The yeast strain BY4741 was transformed with plasmids pGBK-Hisp33-CUP1/DI72-GAL1, pGAD-His92-CUP1 and either pYC-HF, pYC-HF-VPS4, pYC-HF-VTA1 or pYC-HF-SSA1. Additionally yeast was transformed with pGBK-FLAGp33-CUP1/DI72-GAL1 and pGAD-His92-CUP1. Transformed yeasts were pre-grown in SD minimal media with 2% glucose for 20 h at 29°C, then transferred to SD minimal media with 2% galactose for 24 h at 29°C. The cultures were then supplemented with 50 µM CuSO_4_ for 3 h to induce expression of viral proteins. The yeasts were collected by centrifugation and subjected to formaldehyde cross-linking and FLAG purification as described above. The FLAG purified fractions, eluted in SDS-PAGE loading buffer, were treated with phenol/chloroform and ethanol precipitated to recover co-purified RNA. For detection of DI-72(+) RNA, RT reactions were done with SuperScript II (Life Technologies) and primer #22 (GTAATACGACTCACTATAGGGCTGCATTTCTGCAATGTTCC), followed by PCR with primers #1165 (AGCGAGTAAGACAGACTCTTCA) and #927 (TAATACGACTCACTATAGG). For detection of DI-72(−) RNA, the primer used for RT was #18 (GTAATACGACTCACTATAGGAGAAAGCGAGTAAGACAG), followed by PCR with primers #1190 (GGGCTGCATTTCTGCAATG) and #927.

### Analysis of replication in cell free extracts

The yeast strains BY4741 and *vps4Δ::hphNT1* were transformed with plasmids pGBK-FLAGp33-CUP1 and pGAD-FLAGp92-CUP1 [67]. Transformed yeasts were grown and cell free extracts prepared as described [49] except that the cultures were supplemented with 50 µM CuSO_4_ 1.5 h before harvesting, to induce expression of p33 and p92, and incubated at 37°C for 45 min before harvesting. Replicase reactions were carried out as described [49]. RNA protection was tested by adding 1 mM CaCl_2_ and 50 ng micrococcal nuclease at selected time points followed by 15 min incubation and then inactivation of the nuclease by addition of 2.5 mM EGTA. All reactions were incubated for a total time of 3 h.

### Electron microscopic studies

Yeast strains (see Supplementary materials) were pre-cultured from plated single colonies by inoculation in 2 ml of YPG (yeast extract peptone galactose) and shaking overnight at 250 rpm at 30°C. For inducing and maintaining viral replication, yeasts cells were grown for 24 h in YPG at 23°C and shaken at 250 rpm. When OD_600_ was around 2, cells were collected, centrifuged for 5 min at 4000 g, resuspended in TSD reduction buffer (Tris-sulfate DTT, pH 9.4) and either chemically fixed for electron microscopy (see below) or processed for removing the cell wall and obtaining spheroplasts.

For obtaining spheroplasts, yeast cells were incubated for 10 min at room temperature and treated at 30°C with 0.1 µg/µl zymolyase 20T (AMS Biotechnology) in spheroplast medium A (1× yeast nitrogen base, 2% (w/v) glucose, 1× amino acids, 1 M sorbitol, 20 mM TrisCl, pH 7.5) for 5 or 15 min, depending on the yeast strain. After zymolyase treatment, cells were centrifuged for 5 min, at 1000 g and 23°C and washed once with spheroplast medium B (1× yeast nitrogen base, 2% (w/v) glucose, 1× amino acids, 1 M sorbitol) and twice with spheroplast medium A.

For ultrastructural studies, yeast cells and spheroplasts were processed. Compared to whole yeast cells, spheroplasts are well infiltrated with fixatives and resins allowing an optimal visualization of intracellular compartments. Cells were first fixed for 20 min in suspension at room temperature with 8% paraformaldehyde and 1% glutaraldehyde followed by a second fixation step of 1 h at room temperature with 4% paraformaldehyde and 0.5% glutaraldehyde in HEPES (pH 7.4); fixed cells were then processed by conventional embedding in the epoxy-resin EML-812 (Taab Laboratories) following procedures for an optimal preservation of cell endomembranes [65], [69], [70]. Cells were post-fixed for 1 h at 4°C with 1% osmium tetroxide and 0.8% potassium ferricyanide in water, washed with HEPES, and 40 min with 2% uranyl acetate at 4°C. During post-fixation, samples were protected from light. Cells were submitted to dehydration steps for 20 min each with increasing concentrations of acetone (50, 70, 90, and twice in 100%) at 4°C and incubated with acetone-resin (1∶1) with gentle agitation at room temperature. Cells were infiltrated overnight with pure resin for 1 day and polymerized at 60°C for 3 days. Ultrathin (50–70 nm) sections were collected in 300 mesh cooper grids (G300-C3, Taab) with a plastic layer of 0.25% formvar in chloroform. Then, grids were stained for 20 min with saturated uranyl acetate and for 2 min with lead citrate following standard procedures. Samples were studied in a Jeol JEM 1011 electron microscope operating at 100 kv.

For visualization of MT-tagged p33 in cells and immunogold labeling, cells were processed by embedding in the acrylic resin LRWhite following procedures for an adequate preservation of protein epitopes and optimal visualization of small nano-clusters [66]. Spheroplasts were incubated for 75 min with 0.2 mM HAuCl_4_ (SIGMA-ALDRICH) in spheroplast medium A. This treatment builds gold nano-clusters in MT-tagged proteins allowing detection of protein molecules in cells with high sensitivity and at molecular scale resolution [66], [71]. Cells were washed with spheroplast medium A before fixation with 4% paraformaldehyde and 0.2% glutaraldehyde in PHEM (20 mM PIPES, 50 mM HEPES, 20 mM EGTA and 4 mM MgCl_2_, pH 6.9) (1 h at room temperature). Cells were submitted to short dehydration steps of 10 min each in increasing concentrations of ethanol (30, 50, 70, 90 and twice with100%) at 4°C. Spheroplasts were incubated in mixtures of ethanol- LR White acrylic resin (2∶1, 2∶2, 1∶2) with gentle agitation and protected from light and embedded in 100% resin for 24 h. Samples were polymerized for 48 h at 60°C. Ultra-thin sections were collected in 300 mesh Quantifoil holey carbon grids (R 3.5/1 Cu/Rh, Quantifoil Micro Tools) and studied without staining.

For immunogold labeling sections of cells embedded in LR White acrylic resin were processed as described [70]. Briefly, sections were incubated for 6 min with 1% BSA (Bovine serum albumin) in PBS, with primary antibodies diluted in 1% BSA and with secondary antibodies conjugated with 5 or 10 nm colloidal gold particles (from BB International) and diluted in 1% BSA. Dilutions of antibodies were rabbit anti-HA 9110 from ABCAM (1∶200) and mouse anti-dsRNA MAb J2 from English & Scientific Consulting (1∶200). Secondary antibodies were diluted 1∶40.

## Supporting Information

Figure S1 **TEM of stained ultra-thin sections of control BY4741 yeasts.** (A–B) Independent cells, which do not express the tombusvirus replication proteins, are shown in each panel. Characteristic mitochondria (mi), Nuclei (N) and endomembranes (arrows) are distinguished but spherule-like vesicles are absent. Bars, 100 nm.
(PDF)

Figure S2 **Immunogold detection of Vps4-HA in the absence of the tombusvirus replication proteins.** White arrows in A and B point to 10 nm colloidal gold particles bound to anti-HA. Bars, 100 nm.
(PDF)

Materials and Methods S1 Yeast strains and plasmids. Yeast strains used for electron microscopy.
(DOC)
