# Supplementary figures and images for: Noncanonical Role for the Host Vps4 AAA+ ATPase ESCRT Protein in the Formation of Tomato Bushy Stunt Virus Replicase
Source: PLoS Pathog. 2014 Apr 24;10(4):e1004087. doi: 10.1371/journal.ppat.1004087 (PMC3999190; doi:10.1371/journal.ppat.1004087)

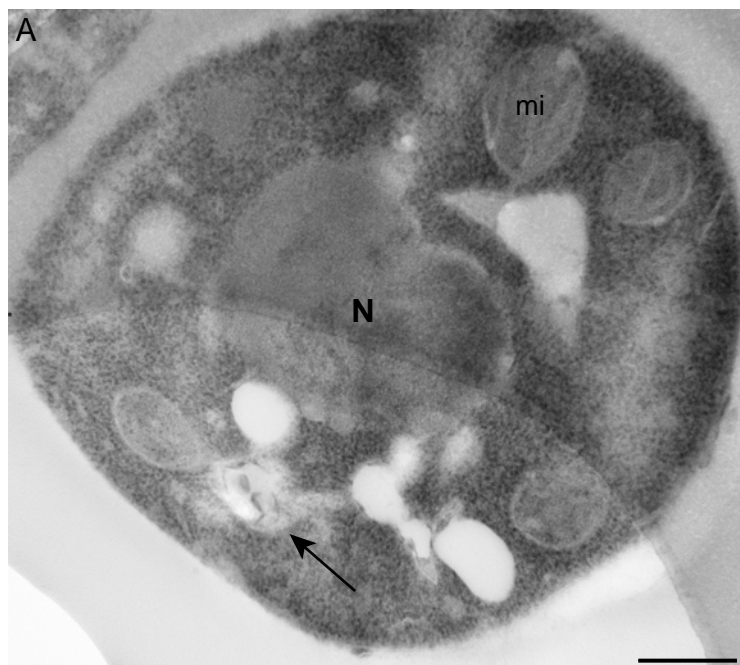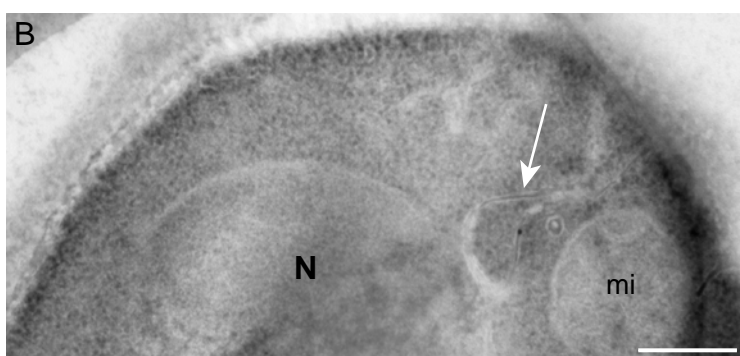

Supplement: Figure S1 — TEM of stained ultra-thin sections of control BY4741 yeasts. (A–B) Independent cells, which do not express the tombusvirus replication proteins, are shown in each panel. Characteristic mitochondria (mi), Nuclei (N) and endomembranes (arrows) are distinguished but spherule-like vesicles are absent. Bars, 100 nm. (PDF) [file ppat.1004087.s001.pdf]

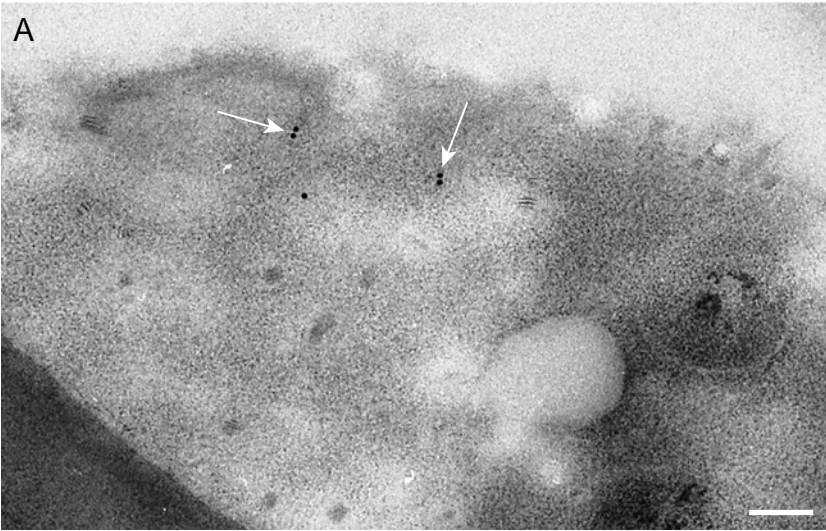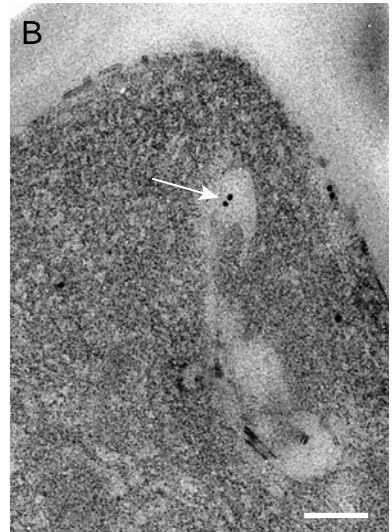

Supplement: Figure S2 — Immunogold detection of Vps4-HA in the absence of the tombusvirus replication proteins. White arrows in A and B point to 10 nm colloidal gold particles bound to anti-HA. Bars, 100 nm. (PDF) [file ppat.1004087.s002.pdf]
