# Supplementary material for: Noncanonical Role for the Host Vps4 AAA+ ATPase ESCRT Protein in the Formation of Tomato Bushy Stunt Virus Replicase
Source: PLoS Pathog. 2014 Apr 24;10(4):e1004087. doi: 10.1371/journal.ppat.1004087 (PMC3999190; doi:10.1371/journal.ppat.1004087)
Supplement: Materials and Methods S1 — Yeast strains and plasmids. Yeast strains used for electron microscopy. (DOC) [file ppat.1004087.s003.doc]

**Supplementary material:**

**Materials and Methods S1**

**Yeast strains and plasmids**. The yeast (*Saccharomyces cerevisiae*) strain BY4741 (*MAT*a *his3*Δ*1 leu2*Δ*0 met15*Δ*0 ura3*Δ*0*) was obtained from Open Biosystems. Yeast strain NMY51 [*MAT*a *his3*∆ *200 trp1*-*901 leu2*-*3, 112 ade2 LYS2*::(*lexAop*)_4_-*HIS3 ura3*::(*lexAop*)_8_-*lacZ ade2*::(*lexAop*)_8_-*ADE2 GAL4*] was obtained from Dualsystems Biotech. The yeast strain *vps4Δ::kanMX* was obtained from Open Biosystems, while *vps4Δ::hphNT1* was made by homologous recombination. For that purpose, the *hphNT1* gene was amplified from plasmid pFA6a-hphNT1 [1] with primers #4783 (GACAAAAATAAAGCAGCATAGAGTGCCTATAGTAGATGGGGTAcgtacgctgcaggtcga) and #3259 (TGTACACAAGAAATCTACATTAGCACGTTAATCAATTGACTAatcgatgaattcgagctc). The PCR product was transformed into BY4741 and recombinant yeasts were selected on YPD-hygromycin plates.

To obtain plasmid pGBK-FLAGp33-CUP1, the plasmid pGBK-FLAG-p33 [2] was digested with *Nco*I and *Pst*I to obtain the FLAG-p33 fragment. Then this fragment was ligated into plasmid pGBK-His33/CUP1 [3] previously digested with *Nco*I and *Pst*I to remove the His_6_-p33 portion. To make pGBK-FLAGp33-CUP1/DI72-GAL1, the CUP1-FLAG fragment was excised from pGBK-FLAGp33-CUP1 by *Bam*HI digestion. The plasmid pGBK-Hisp33-CUP1/DI72-GAL1 [4] was digested with *Bam*HI to remove the CUP1-His_6_ portion and then the CUP1-FLAG fragment was ligated in that place.

ESCRT genes were amplified by PCR from yeast genomic DNA with the following primers: *VPS20* with primers #2051 (GctcCTCGAGggatccATGGGCCAAAAAAGTAGTAAAGTG) and #2052 (CGACCTCGAGTCAGCTAGCGGATAGTAATGCTAAAGGTTCCT), *VPS24* with primers #2291 (GctcCTCGAGggatccATGGATTATATAAAAAAAGCCATATG) and #2174 (CGACCTCGAGTTAGCTAGCGTTTTGCAAAGCTCTCAGCC), *SNF7* with primers #2049 (GctcCTCGAGggatccATGTGGTCATCACTTTTTGGTTG) and #2050 (CGACCTCGAGTCAGCTAGCAAGCCCCATTTCTGCTTGTAGT), *VPS2* with primers #3360 (gccGGATCCATGAGTTTGTTTGAGTGGGT) and #3361 (cggCTCGAGTCAGCTAGCAGTCTGCTTCTTCAAAGTGTTC), *DID2* with primers #3358 (gccGGATCCATGTCACGTAATTCTGCAGC) and #3359 (cggCTCGAGTCAGCTAGCGCCCCTCAATGCTCTTAG) and *VPS4* with primers #2057 (GctcCTCGAGggatccATGAGCACGGGAGATTTTTTAAC) and #2058 CGACCTCGAGCTAGCTAGCGTTACCTTCTTGACCAAAATCTC). The PCR products were digested with *Bam*HI and *Xho*I and ligated into pYES2/NT/C (Invitrogen) digested with *Bam*HI and *Xho*I. The resulting plasmids express His_6_-tagged ESCRT proteins from the *GAL1* promoter. Additionally the digested PCR products were ligated into pPR-N-RE [2] digested with *Bam*HI and *Sal*I.

Plasmid pYES-His-PEX19 has been described elsewhere [5]. Plasmid pYES-SSA1 expresses a His_6_-tagged Ssa1 protein from the *GAL1* promoter [6]. Segments from the *VPS4* gene were cloned into pPR-N-RE after amplification by PCR with the following primers: *vps4_1-100_* with primers #2057 and #4507 (CGGctcgagTTAgctagcAGAGATTTTCTTATTACCACCG), *vps4_101-300_* with primers #4508 (gccggatccCAGGAAGAGGGTGAGGATAATG) and #4509 (CGGctcgagTTAgctagcATCTGGTAATGGTATATATATTC), *vps4_301-437_* with primers #4510 (GCCgctagcggatccTTGGCGGCAAGAACCACC) and #2058, *vps4_1-300_* with primers #2057 and #4509 and *vps4_101-437_* with primers #4508 and #2058. The PCR products were digested with *Bam*HI and *Xho*I and ligated into pPR-N-RE digested with *Bam*HI and *Sal*I. The *Bam*HI and *Xho*I-digested full-length *VPS4* and *vps4_101-437_* products, were also cloned into the centromeric plasmid pYC2/NT/C (Invitrogen), that was digested with *Bam*HI and *Xho*I, or into pGEX-His [4], that was digested with *Bam*HI and *Xho*I.

The plasmids pMALc2x, coding for the maltose binding protein (MBP), pMAL-33c, coding for the MBP fused to the C-terminal half of TBSV p33 (aa 150 to 296) and others coding for MBP fused to portions of TBSV p33c have been described [7]. To produce plasmids expressing MBP fused to p33 portions spanning S1-LR-S2 region, PCR reactions were performed using the following primers: #4974 (gcggatccctgcagGCCCAACGTCGTGACGAAAACGGCACCagactaatctaccagagg) and #4976 (Gccgaagcttctcgagctacgactcctccactccatc) for S1-LR-S2, #4974 and #4977 (Gccgaagcttctcgagctaaatagccaaaggcaatatgac) for S1-LR, #4974 and #4978 (Gccgaagcttctcgagctagtctttgtccatgatctcg) for S1, #4975 (gcggatccctgcagGCCCAACGTCGTGACGAAAACGGCACCatcatggacaaagactgc) and #4976 for LR-S2, #4975 and #4977 for LR. The PCR products were digested with *Pst*I and *Hind*III and cloned into pMALc2x digested with *Pst*I and *Hind*III.

To make plasmids expressing selected host proteins fused to a FLAG tag, *VPS4* and *VTA1* genes were amplified from yeast DNA with primers #2057 and #2058 or #4686 (GCCGGATCCATGGCTAGTAACGCTGCAAG) and #4687 (CGGctcgagTCAgctagcAATAGAATTTAACAGATCCAAC), respectively. The products were digested with *Bam*HI and *Xho*I and cloned into *Bam*HI / *Xho*I-digested pYC-HF [6]. The plasmid pYC-HF-SSA1 was described before [6].

**Yeast strains for electron microscopy.** The yeast strain BY4741 was transformed with a DNA cassette, designed to integrate at retrotransposon sites, coding for the *KanMX4* marker and the His_6_-p92 under control of the *ADH1* promoter and *ADH1* terminator [8], generating BY4741 *ADH1*p-His-p92::*KanMX4*. The metallothionein (MT) [9] coding sequence was amplified with primers #4055 (cggcAGATCTaccatggaccccaactgctcctgc) and #4035 (ccagggatccggcacagcacgtgcacttgtc), digested with *Bgl*II and *Bam*HI and inserted into *Bam*HI-digested pESC-DI72-His33 [5]. The resulting plasmid was used as a template for PCR with primers #2756 (gccgAGATCTgagcgacctcatgctatac) and #3654 (CTAATGAATCCATTTGTTTGTTAATAGTTTGAATGTTTTTAatgcagctggatcttcgag). The PCR product was digested with *Bgl*II and ligated to the *Bgl*II-digested pFA6-*hphNT1* plasmid [1]. The ligation product was used as template for PCR with primers #3653 (CATGGTAGCGCCTGTGCTTCGGTTACTTCTAAAGAAGTCCAAatcgatgaattcgagctc) and #3654. The resulting PCR product, coding for the *hphNT1* marker, the His_6_-MT-p33 under control of *GAL1* promoter and *CYC1* terminator, and DI-72 under control of *GAL10* promoter and *ADH1* terminator, was transformed into BY4741 *ADH1*-His-p92::*KanMX4* to generate the yeast strain DB-614 (BY4741 *ADH1*p-His-p92::*kanMX4*, *GAL1*p-His-MT-p33/*GAL10*p-DI72::*hphNT1*). To delete *VPS4,* the *URA3* gene was amplified from plasmid pCM189 (Euroscarf) with primers #2446 (AATAAGAACTTCATGATATCGAACGATTTAATCGTGGGTTGaggccctttcgtcttcaag) and #2447 (TTTCATGTACACAAGAAATCTACATTAGCACGTTAATCaattgaagctctaatttgtgag). The PCR product was transformed into strain DB-614 and recombinants were selected in plates lacking uracyl. To fuse Vps4p to a C-terminal 6xHA tag, a *6xHA::natNT2* cassette was amplified from plasmid pYM17 [1] with primers #3258 (GAAGCAAGAACAGTTCACTAGAGATTTTGGTCAAGAAGGTAACcgtacgctgcaggtcga) and #3259. The PCR product was transformed into strain DB-614 and recombinant yeasts were selected in YPD plates supplemented with 100 mg/l nourseothicin.

**REFERENCES**

1. Janke C, Magiera MM, Rathfelder N, Taxis C, Reber S, et al. (2004) A versatile toolbox for PCR-based tagging of yeast genes: new fluorescent proteins, more markers and promoter substitution cassettes. Yeast 21: 947-962.

2. Li Z, Barajas D, Panavas T, Herbst DA, Nagy PD (2008) Cdc34p ubiquitin-conjugating enzyme is a component of the tombusvirus replicase complex and ubiquitinates p33 replication protein. J Virol 82: 6911-6926.

3. Jaag HM, Stork J, Nagy PD (2007) Host transcription factor Rpb11p affects tombusvirus replication and recombination via regulating the accumulation of viral replication proteins. Virology 368: 388-404.

4. Barajas D, Li Z, Nagy PD (2009) The Nedd4-type Rsp5p ubiquitin ligase inhibits tombusvirus replication by regulating degradation of the p92 replication protein and decreasing the activity of the tombusvirus replicase. J Virol 83: 11751-11764.

5. Pathak KB, Sasvari Z, Nagy PD (2008) The host Pex19p plays a role in peroxisomal localization of tombusvirus replication proteins. Virology 379: 294-305.

6. Serva S, Nagy PD (2006) Proteomics analysis of the tombusvirus replicase: Hsp70 molecular chaperone is associated with the replicase and enhances viral RNA replication. J Virol 80: 2162-2169.

7. Rajendran KS, Nagy PD (2003) Characterization of the RNA-binding domains in the replicase proteins of tomato bushy stunt virus. J Virol 77: 9244-9258.

8. Kovalev N, Pogany J, Nagy PD (2012) A Co-Opted DEAD-Box RNA Helicase Enhances Tombusvirus Plus-Strand Synthesis. PLoS Pathog 8: e1002537.

9. Diestra E, Fontana J, Guichard P, Marco S, Risco C (2009) Visualization of proteins in intact cells with a clonable tag for electron microscopy. J Struct Biol 165: 157-168.
